# Risk-adjusted perioperative bridging anticoagulation reduces bleeding complications without increasing thromboembolic events in general and visceral surgery

**DOI:** 10.1186/s12871-023-02017-z

**Published:** 2023-02-16

**Authors:** Ida Döhler, Daniel Röder, Tobias Schlesinger, Christian Alexander Nassen, Christoph-Thomas Germer, Armin Wiegering, Johan Friso Lock

**Affiliations:** 1 Department of General, Transplantation, Vascular and Pediatric Surgery, University Hospital of Würzburg, 97080 VisceralWürzburg, Germany; 2 Department of Anesthesiology, Intensive Care, Emergency and Pain Medicine, University Hospital of Würzburg, Würzburg, Germany

**Keywords:** Low-molecular heparin, Atrial fibrillation, Postoperative bleeding, Thromboembolism, Anticoagulation, Bridging

## Abstract

**Background:**

Perioperative bridging of oral anticoagulation increases the risk of bleeding complications after elective general and visceral surgery. The aim of this study was to explore, whether an individual risk-adjusted bridging regimen can reduce bleeding events, while still protecting against thromboembolic events.

**Methods:**

We performed a quality improvement study comparing bridging parameters and postoperative outcomes before (period 1) and after implementation (period 2) of a new risk-adjusted bridging regimen. The primary endpoint of the study was overall incidence of postoperative bleeding complications during 30 days postoperatively. Secondary endpoints were major postoperative bleeding, minor bleeding, thromboembolic events, postoperative red blood cell transfusion, perioperative length-of-stay (LOS) and in-hospital mortality.

**Results:**

A total of 263 patients during period 1 and 271 patients during period 2 were compared. The included elective operations covered the entire field of general and visceral surgery. The overall incidence of bleeding complications declined from 22.1% during period 1 to 10.3% in period 2 (*p* < 0.001). This reduction affected both major as well as minor bleeding events (8.4% vs. 4.1%; *p* = 0.039; 13.7% vs. 6.3%; *p* = 0.004). The incidence of thromboembolic events remained low (0.8% vs. 1.1%). No changes in mortality or length-of-stay were observed.

**Conclusion:**

It is important to balance the individual thromboembolic and bleeding risks in perioperative bridging management. The risk adjusted bridging regimen reduces bleeding events in general and visceral surgery while the risk of thromboembolism remains comparably low.

## Introduction

An aging population leads to a strongly increasing incidence of diseases as atrial fibrillation, mechanic heart valves and thrombosis [1, 2]. This also affects the need for oral anticoagulation (OAC) using warfarin or direct oral anticoagulants (DOACs). Notably, these elderly people increasingly require surgery [3] for age-related comorbidities, such as hernia, diverticulitis or gastrointestinal carcinoma. Recent studies highlighted the effects of perioperative bridging versus non-bridging on thromboembolic events and bleeding complications [4]. Most of these trials showed that placebo was non-inferior to low molecular weight heparin (LMWH) bridging concerning thromboembolic events, but they revealed an increased risk of bleeding in bridged patients [5]. A reduced use of LMWH bridging has been reported since publication of the BRIDGE-trial [6]. However, general and visceral surgery made up only a small proportion within the recent study populations.

There is a need for further evidence on perioperative OAC bridging and postoperative bleeding complications in general and visceral surgery, in particular since postoperative hemorrhage is one of the major complications in this field [7]. Our initial study reported that especially full-therapeutic-dose LMWH strongly increases the risk of postoperative bleeding complications. Based on this experience, we developed a less aggressive and individually risk-adjusted bridging regime [8].

The aim of this quality improvement study was to evaluate the local implementation of the new bridging regimen concerning its efficacy to reduce bleeding complications and its safety aspects concerning thromboembolic events.

### Patients and methods

This quality improvement study was conducted in a German 1,500-bed university hospital (University hospital Würzburg) comparing perioperative bridging anticoagulation before (period 1) and after implementation (period 2) of a new local bridging regimen. The manuscript was prepared according to the Standards for Quality Improvement Reporting Excellence (SQUIRE) guidelines [9]. The retrospective analysis was approved by the local institutional ethical review board (Ethik-Kommission der Julius-Maximilians-Universität Würzburg; Ref. 20,210,505 03) without need for informed consent because only medical records were analyzed anonymously.

### Patients

We chose to review a three-year-period following local implementation of the new bridging regimen (2017–2020) to evaluate changes in bridging practice (period 2). The data of our previously published patient cohort receiving bridging during January 1, 2011 and December 31, 2014 [8] were applied as comparator (period 1).

The data for were collected retrospectively from our hospital electronic database. Main sources of information were patient charts, documentation of preoperative assessment and anesthesia, surgical reports, laboratory findings and discharge documents. A collective of 791 cases for period 2 were identified by the center for medical computing at our clinic. Selection criteria for these automatically generated case lists were in-hospital surgery and one of the following International Statistical Classification of Diseases and Related Health Problems (ICD) 10 diagnosis codes: Z92.1 (oral anticoagulation), D68.5-D68.9 (thrombophilia) and I48.0-I48.3 (atrial fibrillation). Subsequently, these patients were screened for the following inclusion and exclusion criteria: Inclusion criteria were age ≥ 18 years, American Society of Anesthesiologists (ASA) physical status classification < 5, elective general or visceral surgery and OAC. Exclusion criteria were emergency surgery, vascular and bariatric surgery or endoscopic procedures. Furthermore, patients were excluded who suffered from erosive bleeding, intraoperative massive transfusion or sepsis, which made a transfusion necessary. Duplicates and incomplete datasets were also excluded. Finally, a collective of 271 patients receiving surgery after implementation of the new bridging regimen (period 2) were available.

The risk of thromboembolism was categorized for the statistical analysis according to the guidelines of the German Society of Cardiology [10], the European Society of Cardiology [11] and the American College of Chest Physicians Evidence-Based Clinical Practice Guidelines on perioperative management of antithrombotic therapy [12]. The CHADS_2_ score, validated by Gage et al. [13], was used to estimate the risk of stroke in atrial fibrillation (low risk of thromboembolism: CHADS_2_ score 0–2, venous thrombosis > 12 months, mechanical aortic valve without risk factors; moderate risk: CHADS_2_ score 3–4, venous thrombosis > 3 months, mechanical aortic valve with risk factors or biological aortic valve; high risk: CHADS_2_ score 5–6, venous thrombosis or cerebral ischemia ≤ 3 months, relevant thrombophilia, mitral valve replacement). The procedure-specific risk of bleeding was divided into five categories [14] (e.g. minimal risk: skin incision/ biopsy; mild risk: ileostomy reversal, inguinal hernia, hemithyroidectomy, laparoscopic cholecystectomy; moderate risk: hemicolectomy, large incisional hernia, open cholecystectomy, thyroidectomy, gastric wedge resection; major risk: rectum resection, hemihepatectomy, gastrectomy; critical risk: extended hemihepatectomy; extended gastrectomy; pancreas head resection). Finally, we verified the adherence to the internal checklist by the hospital staff by comparing the LMWH dosing the patient actually received with the bridging scheme recommended by the new regimen.

### Perioperative management of anticoagulation

Perioperative bridging in period 1 was performed as previously described, with full-therapeutic-dose LMWH bridging (1 mg/kg bodyweight enoxaparin b.i.d) for all patients on OAC with a moderate or high thromboembolic risk [8]. The new bridging regimen was developed in a multidisciplinary approach including experts on the field of surgery, anesthesiology, cardiology and hemostaseology and implemented during 2017 [8]. After finalization of the bridging regimen, the internal training was performed on May 8^th^ 2017. The bridging regimen was implemented as an individual preoperative checklist during preoperative work-up and internally distributed. During period 2, full-therapeutic-dose LMWH was only recommended for patients with high thromboembolic risk. Half-dose LMWH was recommended for moderate thromboembolic risk. Patients with low thromboembolic risk were recommended general thrombosis prophylaxis (prophylactic dose LMWH. The LMWH dose during bridging was adjusted in obesity, age > 75 years and chronic renal insufficiency eGFR < 60 ml/min. A preoperative interruption of anticoagulation (mostly LMWH) > 24 h and postoperatively > 24 h in therapeutic-dose were recommended. The new bridging regimen also allowed DOACs without LMWH bridging.

### Study endpoints

The primary endpoint of the study was overall incidence of postoperative bleeding complications during 30 days postoperatively. Secondary endpoints were major postoperative bleeding, minor bleeding, thromboembolic events, postoperative red blood cell transfusion, perioperative length-of-stay (LOS) and in-hospital mortality. Major postoperative bleeding was defined as a postoperative event leading to a decrease of hemoglobin level > 2 g/dl AND requiring surgical or radiological intervention for bleeding control, OR transfusion of ≥ 2 units of packed red blood cells as defined by the International Society on Thrombosis and Haemostasis [15]. Minor postoperative bleeding events were defined as clinically apparent events, that were explicitly documented within the patient records, e.g. hematoma or blood loss via drainages leading to a clinical significant decrease of hemoglobin concentration (> 2 g/dl). Incidental radiological findings without clinical symptoms such as small hematomas were not considered bleeding events. Intraoperative blood loss was not taken into account due to lack of standardized intraoperative measurement and the consecutive low data quality. Thromboembolic events were defined as postoperative diagnosis of stroke, transient ischemic attack, arterial embolism, myocardial infarction, deep vein or pulmonary embolism.

### Sample size calculation

A sample size estimation was performed based on our previous study reporting an overall incidence of postoperative bleeding complications of 22.1% within the bridging group of period 1 [8]. We assumed that the new bridging regimen would strongly decrease the application of full-therapeutic-dose LMWH leading to a reduction of postoperative bleeding events by 50% within period 2. To substantiate a significant group difference with a 2-sided test at a level of α = 0.05, 236 patients (> 90% Power) were required per group.

### Statistical analysis

Descriptive analyses are reported as the mean and 95%-confidence interval of the mean, unless otherwise noted*.* P values were calculated by T test, Welch test, χ^2^-test or Fisher’s exact test according to data distribution. The level of significance was 0.05 (two-sided). IBM SPSS Statistics, version 26 (International Business Machines Corporation, Armonk, NY) was used to perform the analysis.

## Results

### Baseline characteristics

Two hundred sixty-three consecutive patients during period 1 and two hundred seventy-one consecutive patients during period 2 were analyzed. There were no significant changes in terms of age, sex, body mass index (BMI), indication of OAC, risk of thromboembolism, risk of bleeding and procedure-specific risk of bleeding within the study periods (Table 1) OAC was indicated predominantly due to atrial fibrillation (83% vs. 76%). Thromboembolic risk was low in the majority of patients (52% vs. 56%) while the medical bleeding risk was high (66% vs. 69%). The surgical specific risk of bleeding was predominantly mild (46% vs. 50%) or moderate (34% vs. 32%).

**Table 1:** Patient characteristics and preoperative risk factors

Characteristic, n (%)		Period 1 *n* = 263	Period 2 *n* = 271	*p* value^**1**^
Age, y, mean (95%CI)		71 (70–72)	73 (71–74)	0.09
Sex, male, %		63.5	63.1	0.92
Body mass index [kg/m^2^], mean (95%CI)		28 (27–28)	28 (27–28)	0.53
ASA score > 2		207 (79)	182 (71)	0.04
Bridging indications	Atrial fibrillation	217 (83)	206 (76)	0.064
	Past thromboembolic events	31 (12)	25 (9)	0.33
	Heart valve replacement	22 (9)	21 (8)	0.79
	Thrombophilia	9 (3)	16 (6)	0.18
Risk of thromboembolism	low	136 (52)	152 (56)	0.57
	moderate	75 (29)	68 (25)	
	high	52 (20)	51 (19)	
Risk of bleeding	HAS-BLED score, mean (95%CI)	3.0 (2.9–3.2)	3.1 (2.9–3.2)	0.61
	high bleeding risk [HAS-BLED score > 2]	173 (66)	185 (69)	0.54
Procedure specific risk of bleeding	minimal (category 1)	0	0	0.86
	mild (category 2)	121 (46)	133 (50)	
	moderate (category 3)	90 (34)	87 (32)	
	major (category 4)	40 (15)	37 (14)	
	critical (category 5)	12 (5)	14 (5)	
Cardio-vascular risk factors	Hypertension	219 (83)	213 (79)	0.17
	Congestive heart failure	88 (34)	52 (19)	< .001
	Coronary heart disease/arterial occlusive disease	89 (34)	67 (25)	0.02
	Chronic renal insufficiency ≥ stage III (GFR < 60 ml/min)	97 (37)	85 (31)	0.18
	Diabetes mellitus	79 (30)	75 (28)	0.55

^1^*p* values of continuous outcomes were calculated by Welch test, p values of categorical outcomes were calculated by a two-sided χ^2^-test or, in case of categories with less than 5 subjects, by Fisher’s exact test

Regarding the comorbidities, the study periods did not differ for hypertension, diabetes or chronic renal insufficiency. In contrast, a higher incidence of coronary heart disease or arterial occlusive disease and congestive heart failure were recorded during period 1.

The type of OAC significantly changed during the study periods: While phenprocoumon was predominant during period 1 (76%), DOACs became common during period 2 (53%; *p* < 0.001). During period 1, only rivaroxaban was available as DOAC, while during period 2 rivaroxaban (41%), apixaban (37%), edoxaban (19%) and dabigatran (9%) were applied.

The analyzed surgical procedures were predominantly colorectal, hernia and gall bladder surgery, but also included thyroid, upper gastrointestinal, hepatic, pancreatic and other procedures (Table 4). No significant differences between the study periods were observed. However, there was a higher percentage of oncological surgery within period 2 (25.1% vs. 33.6%; *p* = 0.031). The majority of patients received abdominal surgery by laparotomy (40% vs. 35%) or laparoscopy (22% vs. 23%; *p* = 0.76).

### Changes in perioperative bridging

The percentage of patients receiving too aggressive bridging, meaning full-therapeutic dose LMWH in low or medium risk patients strongly decreased from 60.8% to 32.8% (*p* < 0.001) during period 2. Thus, the distribution of perioperative LMWH dosing significantly changed in period 2 (Table 2). Despite the medicinal recommendation the majority of patients on DOACs received postoperative bridging in both. However, 86 patients on DOACs (60%) were continued orally prior discharge during period 2. While preoperative interruption of anticoagulation for ≥ 24 h was observed in 41% of all cases during the period 1, this proportion almost doubled during period 2. However, the full adherence to the new regime was only 54% during period 2, and 32.8% of patients still received a too aggressive perioperative bridging in reflection of their individual thromboembolic risk.

**Table 2:** Changes in perioperative bridging

**Characteristic**, *n* (%)	**Period 1** *n* = 263	**Period 2** *n* = 271	***p* value**
Bridging			
full-dose LMWH^1^	189 (72)	92 (34)	< 0.001
half-dose LMWH^1^	26 (10)	64 (24)	
too aggressive^2^	160 (60.8)	89 (32.8)	< 0.001
despite DOAC^3^	18 (78.2)	79 (54.8)	0.034
Preoperative interruption of anticoagulation ≥ 24 hours^4^	107 (41)	214 (79)	< 0.001
Normal thrombosis prophylaxis^5^	48 (18)	70 (26)	< 0.001
No perioperative LMWH application	0	27 (10)	< 0.001
Restart of OAC before discharge	3 (1.1)	86 (32)	< 0.001

^1^LMWH, low molecular weight heparin

^2^Defined as full-dose LMWH in patients at low or moderate risk of thromboembolism

^3^DOAC, direct oral anticoagulants. Peri-interventional bridging is not recommended in the medicinal products information of those drugs

^4^Including preoperative LMWH bridging or DOAC application

^5^Normal prophylactic dose of LMWH as for general patients undergoing surgery

#### Postoperative complications and outcome

The overall incidence of bleeding complications declined from 22.1% during period 1 to 10.3% in period 2. Both major and minor bleeding complications were reduced by more than 50% during period 2 (Table 3). Accordingly, the number of patients requiring postoperative red blood cell transfusions also dropped by 50%. The reduction of postoperative bleeding was homogenously throughout the level of surgical bleeding risk, but only mild risk of bleeding was statistically significant (Table 4). Concerning the operated organ, reduction of bleeding was observed in all categories except thyroid surgery.

**Table 3:** Postoperative complications and patient outcome

Characteristic, *n* (%)		Period 1 *n* = 263	Period 2 *n* = 271	*p* value^**1**^
Postoperative bleeding^2^		58 (22.1)	28 (10.3)	< 0.001
within DOAC^3^		5 (21.7)	12 (8.3)	0.048
major		22 (8.4)	11 (4.1)	0.039
minor		36 (13.7)	17 (6.3)	0.004
Red blood cell transfusion		23 (8.7)	12 (4.4)	0.044
Thromboembolism		2 (0.8)	3 (1.1)	1
Clavien-Dindo^4^	Grade IIIa—V	61 (23.2)	66 (24.4)	0.753
	Grade IVa—V	18 (6.8)	16 (5.9)	0.656
Reoperation		40 (15.2)	42 (15.5)	0.926
In-hospital mortality		4 (1.5)	9 (3.3)	0.262
LOS, d, mean (95%CI)		10.5 (9.1–11.8)	10.0 (8.7–11.3)	0.63

^1^*p* values of continuous outcomes were calculated by Welch test, *p* values of categorical outcomes were calculated by a two-sided χ^2^-test or, in case of categories with less than 5 subjects, by Fisher’s exact test

^2^according to the International Society on Thrombosis and Haemostasis

^3^percentages only within the group of patients receiving DOAC

^4^according to Clavien-Dindo classification

^5^postoperative length-of-stay

**Table 4:** Surgical procedures and specific bleeding risk by organ

	***n* (%)**	**Procedure specific risk of bleeding**^**1**^**, %**				**Postoperative bleeding, *n* (%)**	
		**Mild**	**Moderate**	**Major**	**Critical**	**Period 1**	**Period 2**
Surgery by organ							
Colorecta**l**	151 (28)	33	48	19	1	16 (19)	7 (10.4)
Hernia	101 (19)	86	14	-	-	12 (23.1)	7 (14.3)
Gallbladder	62 (12)	87	13	-	-	9 (28.1)*	1 (3.3)*
Thyroid	50 (9)	38	62	-	-	4 (14.8)	4 (17.4)
Lymphatic	30 (6)	7	90	3	-	2 (11.1)	0
Liver	28 (5)	4	11	71	14	5 (31.3)*	0*
Upper GI	38 (7)	13	16	61	11	3 (20)	3 (13)
Pancreas	19 (4)	-	-	16	84	2 (28.6)	2 (16.7)
Other	55 (10)	67	27	4	2	5 (41.7)*	4 (9.3)*
**Postoperative bleeding, *n* (%)**							
Period 1		27 (22.3)*	17 (18.9)	11 (27.5)	3 (25)		
Period 2		10 (7.5)*	10 (11.5)	6 (16.2)	2 (14.3)		

^1^Percentages for each organ. * *p* value < 0.05, calculated by two-sided Mantel-Haenzsel χ^2^-test

The incidence of postoperative thromboembolic events remained unaltered at approx. 1% during the study. However, despite the reduction of bleeding complications, no changes on overall postoperative morbidity and mortality, as well as LOS were observed during period 2.

## Discussion

In this quality improvement study we observed a significant reduction of postoperative bleeding complications after implementation of a new risk-adjusted bridging regimen. Both major and minor bleeding events, as well as postoperative transfusions were reduced by 50% without an increase of thromboembolic events. The risk-adjusted bridging regimen lead to an increasing rate of individually adjusted bridging dosage and perioperative interruption of anticoagulation or LMWH.

Despite multiple trials on bridging during OAC interruption, only few evidence exists concerning the efficacy and risks of perioperative bridging in general and visceral surgery. The percentage of those surgical procedures was relatively small in those studies, e.g. 2.3% BRIDGE-trial [16]; 4% PROSPECT trial [17]; 14.5% PERIOP2 [18]. In this study, a wide range of different general and visceral surgical procedures with a relevant bleeding risk (> 50% with at least moderate risk) were included. The comparison of patient characteristics between the study periods revealed some differences at ASA score and the incidences of congestive heart failure and arterial disease. In addition there was a strong increase of DOAC usage in period 2 (from 9 to 53%). Interestingly, the reduction of bleeding events was even stronger within the DOAC patients thus the changes within OAC did clearly not cause the reduction of bleeding events during period 2. As both periods were similar concerning the OAC indication, the risk of thromboembolism and the procedure specific risk of bleeding, we chose not to apply propensity score matching but to compare these real world data.

The implementation of the new regime significantly changed our local bridging practice. The previously reported high incidence of therapeutic-dose LMWH was reduced by more than 50% while the preoperative cessation LMWH for at least 24 h doubled. These two main changes led to the strong reduction of postoperative bleeding events. Also, we found that, contrary to our guideline, patients considered to be at low or moderate risk of thromboembolism receiving therapeutic dosage of LMWH. Thus continuous internal training is required to achieve sufficient adherence to the bridging regime.

Furthermore, more patients underwent early postoperative resumption of OAC in period 2. This might be explained by the increasing DOAC prescription over the last years, which eases the perioperative management. This raises the question, if LMWH bridging will have any role in future, since the PAUSE study proved a very low risk of bleeding and arterial thromboembolism in patients on DOAC for atrial fibrillation [19]. This very simple option seems favorable in many interventions requiring interruption of OAC. However, some issues remain especially in visceral surgery. Only 28% of the surgical procedures analyzed in the PAUSE-study were gastrointestinal or general surgical interventions. All types of major gastrointestinal surgery might interfere with DOAC resorption, especially during the initial postoperative days. This might have an impact on the risk of thromboembolic events. Nevertheless, no study has yet demonstrated that DOACs do not work after visceral surgery. LMWH bridging could maintain low risk of thromboembolic events while not increasing bleeding risks in case of revision surgery, avoiding antidotes of DOACs which are expensive and not available in all hospitals. Besides, there still are OAC indications, where DOACs have not been approved, as for example heart valve replacement. Also, the evidence base in perioperative handling of DOACs still needs to be strengthened, as most studies on perioperative DOAC handling only compare different DOAC agents or DOACs vs. warfarin [19, 20].

The present study confirms the overall increase of bleeding complications during bridging that has been described in several meta-analyses [5, 21]. Although our incidence of bleeding complications declined after implementation of the risk-adjusted regimen, it remained above our previous reported incidence in patients without OAC (10.3% in period 2 vs. 6.1% control group of period 1 [8]). Thus risk-adjusted bridging with LMWH reduces the burden of bleeding complications but it remains somewhat increased. A recent meta-analysis has indicated a somewhat lower risk by LMWH bridging in comparison to heparin in non-cardiac surgery, but missing statistical significance [22].

The general necessity of perioperative bridging has been challenged by two randomized controlled trials demonstrating no benefit concerning the incidence of thromboembolism from LMWH bridging in patients on warfarin OAC [16, 18]. However, there is yet no consensus whether high risk patients might still profit from LMWH bridging and individual management is still recommended [5, 21, 23]. A single study on bridging in abdominal malignancy surgery reported no increase of bleeding complications or thromboembolism by heparin bridging [24]. In this retrospective analysis, however, only prophylactic-dose heparin was applied and the rate of thromboembolism was relatively high in the bridging group (4.1% vs. 1.1% in period 2 of our study). Therefore our individual risk-adjusted bridging regimen could provide a practical and safe guidance until further evidence emerges. It somewhat balances the bleeding and thromboembolism risks and results might be further improved by increasing internal guideline adherence.

Several limitations of this study require further discussion: Firstly, patients considered at low or moderate risk of thromboembolism received therapeutic dosage of LMWH during period 1 in contrast with guidelines recommendation. Despite the reduction of bleeding complications and fewer transfusion, no further effect on postoperative morbidity and mortality could be observed. Thus optimizing perioperative management of OAC had no strong effect on postoperative outcomes in these elderly and comorbid patients. No determination of intraoperative blood loss or changes in postoperative hemostasis were part of this study and could not be analyzed.

Furthermore, no clear conclusion concerning the risk of thromboembolism can be derived from this analysis focusing on bleeding complications. Further investigation is required to determine whether internal guideline adherence can be improved and if this may further reduce bleeding complications and if this might impact incidence of thromboembolism.

The increasing application of DOACs lead to an imbalance within the two groups. However, the observed reduction of bleeding complication had not been the result of more DOACs. The majority of DOAC patients underwent perioperative bridging in contrast to the medicinal products information.

Finally, we were not able to specify if patients with an only temporary OAC-derived risk of bleeding (e.g. for thrombosis or percutaneous coronary intervention) were delayed for elective surgery. In those patients, surgery should be postponed if possible as surgery and oral anticoagulation are major criteria for a high bleeding risk [25, 26].

## Conclusion

Balancing thromboembolic and bleeding risks during perioperative management of elderly patients receiving OAC is of high clinical relevance to avoid major sequela. Individual risk-adjusted bridging effectively reduces bleeding complications in general and visceral surgery while the risk of thromboembolism remains comparable. Further research concerning the general necessity of warfarin bridging and the perioperative handling of DOACs is required to provide optimal perioperative management for all OAC indications.

## Data Availability

The datasets used and analyzed during the current study are available from the corresponding author on request.
